# Psychological Functioning of Patients Undergoing Oral Surgery Procedures during the Regime Related with SARS-CoV-2 Pandemic

**DOI:** 10.3390/jcm9103344

**Published:** 2020-10-18

**Authors:** Dorota Pylińska-Dąbrowska, Anna Starzyńska, Wiesław Jerzy Cubała, Karolina Ragin, Daniela Alterio, Barbara Alicja Jereczek-Fossa

**Affiliations:** 1 Oral Surgery Department, Medical University of Gdańsk, 80-211 Gdańsk, Poland; karolina.ragin@gumed.edu.pl; 2 Department of Adult Psychiatry, Medical University of Gdańsk, 80-211 Gdańsk, Poland; wieslaw.cubala@gumed.edu.pl; 3 Department of Oncology and Hemato-Oncology, University of Milan, 20122 Milano, Italy; daniela.alterio@ieo.it (D.A.); barbara.jereczek@ieo.it (B.A.J.-F.); 4 Division of Radiotherapy, IEO European Institute of Oncology, IRCCS, 20141 Milan, Italy

**Keywords:** dental anxiety, pandemic, dentistry, oral surgery, dental care, SARS-CoV-2, COVID-19

## Abstract

The coronavirus pandemic has become a huge global challenge medically, economically and psychologically. The COVID-19 pandemic shows that the population can experience general psychological distress. The sanitary regime in dental offices and lack of vaccine for coronavirus may have an impact on the level of dental anxiety among patients undergoing oral surgery procedures. A clinical study was conducted between November 2019 and September 2020. A total of 175 patients (*n* = 175) were enrolled in the research. The aim of the study was to assess the attitude of patients towards the new situation related to the reduced availability of dental offices providing oral surgery procedures. The level of anxiety associated with surgical intervention was measured using a self-made COVID-19 questionnaire and the MDAS scale. The ED-5Q questionnaire and EQ-VAS scale were also used in this research. The study showed that 21.9% of respondents presented with increased anxiety about a dental visit compared with the time before the pandemic. This epidemiological situation has led to an overwhelming increase in moderate dental anxiety (M: 11.4) among patients undergoing oral surgery procedures. The quality of patients’ health (EQ-VAS) related to the impact of the coronavirus pandemic and the quarantine decreased by 10 percentage points. Oral surgeons should be prepared for more anxious patients in dental offices during the pandemic.

## 1. Introduction

The outbreak of the coronavirus pandemic (SARS-CoV-2), which started in December 2019 in Wuhan, China, has become a huge global challenge medically, economically and psychologically [1]. On the 30 January 2020, the World Health Organization (WHO) declared the COVID-19 outbreak a global health emergency. At the end of September 2020, the WHO reported that more than 23 million people had been infected worldwide and 800,000 deaths had been caused by the SARS-CoV-2 infection [2,3]. The number of infected people around the world is still rising. The limited knowledge of COVID-19 and overwhelming news delivery may lead to anxiety and fear in the public.

Three main mechanisms of dental anxiety are hypothesized. One of them is based on patients’ own experiences (mostly traumatic experiences from the past) and the other two result from external factors, such as negative-biased information and observations of negative behavior during dental treatment [4,5,6]. In time, it may transpire that we will also have to deal with another mechanism linked to the difficulty in finding a dental office which provides surgical services during pandemic lockdown and in accordance with the pandemic-related regime. These are factors we did not have to consider in this study. Depending on the examined population and the assessment tools, 2.5–20% of people experience dental anxiety [7,8].

The population at large may experience disappointment, stress and irritability when in isolation [9]. Psychologists from China examined the general population during the initial stage of the COVID-19 pandemic. They found that 53.8% of the respondents rated the psychological impact of the outbreak as moderate or severe, 16.5% reported moderate to severe depressive symptoms, and 28.8% reported moderate to severe anxiety symptoms [10]. Another study, which included more than 50,000 people in China during the coronavirus pandemic, showed that about 35% of people experienced psychological distress [11].

Peloso et al. reported that the pandemic has a considerable impact on dental appointments and anxiety in patients. There was an association between patients’ attitudes towards the pandemic and their enthusiasm to attend a dental appointment. The author reported that at the beginning of the pandemic 28.6% of interviewees reported experiencing anxiety. Their concerns were associated with the risk of getting infected and transmitting the disease to family members [9].

There are some studies on the psychological influence of the COVID-19 pandemic on patients in different medical sectors [11,12,13] yet, there are no studies on patients undergoing oral surgery procedures in outpatient dental surgeries [5]. Assessing the severity of anxiety in this group of patients may contribute to the optimization of the treatment process [12].

The aim of this study was to compare the psychological functioning of patients undergoing dental surgery procedures before and after the outbreak of the pandemic.

## 2. Experimental Section

This study was approved by the Independent Research Ethics Committee of the Medical University in Gdańsk, Poland (NKBBN/366/2016).

A clinical study involving 175 (*n* = 175) patients was carried out. The patients were divided into three groups: those undergoing a surgery before the outbreak of the pandemic, those during the most severe restrictions after the COVID-19 outbreak, and those after the lifting of the most severe restrictions. The patients were consecutively recruited at the Department of Oral Surgery at the Medical University of Gdańsk. The patients’ visits took place at the Oral Surgery Department from November 2019 to September 2020. In 47 instances, the procedures were performed before the SARS-CoV-2 reached Poland (first confirmed case–4 March 2020). At the time, planned surgeries were performed. Patients were given routine verbal information on the course of treatment and the post-operative indications. All verbal information was standardized and presented by the same dental surgeon. During the time of pandemic restrictions 128 (*n* = 128) patients were treated. They received the same verbal information as the group treated before the pandemic and the procedures were performed by the same dentist. This group was divided into two subgroups. The patients from the first subgroup (*n* = 57) underwent the surgeries at the time of the most severe restrictions-between 4 March 2020 and 31 May 2020. The second subgroup (*n* = 71) was admitted after the most severe restrictions were lifted in Poland (31 May 2020) [2].

All examined patients (175) filled in a questionnaire on their sex, age, place of residence and presence or lack of symptoms related to COVID-19. The procedures to which the patients were subjected included tooth extractions, surgical tooth extractions, abscess drainage and drain removals (Table 1). All patients gave their written informed consent for the study. Fear of coronavirus in the group operated on after the outbreak was measured using a custom-built questionnaire consisting of ten statements about COVID-19 (Table 2). A Likert scale with five sorted categories from 1 (definitely no) to 5 (definitely yes) was used here. Patients undergoing surgery before the outbreak of the pandemic did not answer questions about COVID-19. Questionnaires related to the EuroQol 5D Quality of Life Self-esteem, which consists of two parts-EQ-5D (Five Questions of EuroQol 5D Quality of Life Self-esteem Questionnaire) and EQ-VAS (Visual Analogue Scale of EuroQol 5D Quality of Life Self-esteem Questionnaire), and the MDAS (Modified Dental Anxiety Scale) were completed by all 175 patients [14,15,16,17,18]. The participants had a doctor’s help if they had any doubts regarding questions or answers. Patients older than 18 years of age who agreed to participate in the study were considered eligible.

### 2.1. MDAS: Modified Dental Anxiety Scale 

The answers are: calm (1 point), a bit nervous (2 points), nervous (3 points), very nervous (4 points), and extremely nervous (5 points). Points are added up, and the results range between 5 and 25 points. A score of 5 indicates no anxiety, 6–10 a low level of anxiety, 11–14 a moderate level of anxiety and 14–18 a high level of anxiety. A result of more than 19 points indicates an extraordinarily strong level of anxiety, entitling the patient to be included in the group of people suffering from dentophobia. The use of the MDAS questionnaire with each patient before the commencement of dental treatment allows for a simple and objective assessment of the occurrence and severity of anxiety [14,15,16,17].

### 2.2. ED-5Q and EQ-VAS

The ED-5Q questionnaire describes function and quality of life in five dimensions: mobility, self-care, usual activities, pain or discomfort, and anxiety or depression. The EQ-VAS is a visual analogue scale on which the patient is assessing their health on a scale from 0 (worst imaginable health condition) to 100 (best imaginable health condition) [18].

### 2.3. Statistical Analysis

The collected data were subjected to statistical analysis using the STATISTICA 13.1 (StatSoft Inc., Tulsa, OK, USA, serial number JPZ0097539310ARACD-1) licensed by the Medical University of Gdańsk. The quantitative statistical analysis included the chi-square test. For this purpose, the following parameters have been calculated: values mean (M), median (Me), standard deviations (SD), minimum (MIN) and maximum (MAX) values. The Mantel–Haenszel test has also been used. In addition, in several cases where groups had insignificant numbers, Fisher’s exact test was used. Additionally, the non-parametric Mann–Whitney test and the non-parametric Kruskal–Wallis test, supplemented by post-hoc tests, were implemented. The test results were considered significant when *p* < 0.05. Cronbach’s alpha test for the COVID-19 questionnaire was 0.787, for the MDAS was 0.899 and for the ED-5Q was 0.558. The power calculation for the MDAS was −0.03, for the ED-5Q it was −0.18 and for the EQ-VAS it was 0.2. 

## 3. Results

### 3.1. COVID-19 Questionnaire Results

In total, 128 patients completed the COVID-19 questionnaire. Patients undergoing surgeries before the outbreak of the pandemic did not answer these questions. Summary results for affirmative answers are shown in Table 2. Statistical analyses for all the respondents are shown in Table 3 and Table 4. In total, 35.2% of respondents were concerned about the outbreak of the coronavirus pandemic. Half of the examined patients followed all of the news on coronavirus. A total of 50.8% of those surveyed believed that this virus is much more dangerous than seasonal flu. Every sixth respondent was concerned that friends or family would be infected. In total, 83.6% of the examined patients felt safe in a dentist’s office, when they saw a high level of medical staff protection. Every fifth (21.9%) respondent reported that a dental visit made them feel more anxious than before the pandemic.

Comparing patients undergoing surgeries at the time of high restrictions with patients operated on after these restrictions were lifted, statistical significance (*p* < 0.05) was found in questions 8 and 10. Statistical analyses according to the time of admittance are shown in Table 5. A total of 80.7% of patients from the first group admitted that they had tried to cope with pain using home methods before going to a dental appointment, while in the second group the percentage was almost 59.2%. Every seventh respondent operated on at the time of high restrictions expressed concern that friends or family would be infected by SARS-CoV-2, while only every fifth patient gave the same statement after the highest restrictions were lifted (Table 2).

### 3.2. MDAS Results

Of all surveyed, 10.1% did not report any anxiety related to the dental visit. Low levels of anxiety were reported by 39.1% of patients. A moderate level of anxiety was reported in 21.1% cases. Every fifth respondent showed a high level of dental anxiety and 8.6% of patients were extremely anxious. There were no statistically significant differences for different sex or age group categories. The average result of MDAS was 11.4, which means that the examined group is characterized by a moderate level of anxiety (Table 6).

The number of low or moderate anxiety responses according to the MDAS scale was highest in the group of patients operated on after the outbreak of the pandemic. The results show that, after the introduction of restrictions related to the pandemic, the number of patients reporting medium, high or extreme levels of anxiety increased. There was no correlation found between the groups in terms of place of residence, type of procedure, history of anxiety or the time of admittance.

### 3.3. ED-5Q Results

The subjects whose treatment was carried out during the pandemic were characterized by a significantly higher incidence of signaling somatic symptoms and helplessness resulting from being ill (U = 2.286.500; *p* < 0.05). At the same time, the respondents operated on after the outbreak of the pandemic assessed the quality of their health significantly more negatively than those operated on before the introduction of the severe restrictions related to COVID-19 (U = 2227.500; *p* < 0.01). It was determined that there were significant differences between the three groups. Therefore, the multiple comparison procedure was performed using the *Z*-test. The *H* test showed differences between the groups, but the *Z*-test did not confirm these differences. The mean ranks (R) show that in the pre-pandemic group the ED-5Q score was lower than that of the patients undergoing surgery after the outbreak of the pandemic (Table 6).

### 3.4. EQ-VAS Results

The respondents operated on after the outbreak of the pandemic considered their health to be significantly worse when compared with those operated on before the introduction of restrictions related to COVID-19 (U = 2227.500; *p* < 0.01). The quality of patients’ health (EQ-VAS) related to the impact of the coronavirus pandemic was approximately 72, which was 10 points less than before the pandemic (Table 6).

### 3.5. Results and Comorbidities

For all the respondents, a relationship between the number of comorbidities and ED-5Q was found. The results were analyzed according to the types of restrictions effective at the time. The strongest relationship between ED-5Q and the number of diseases was found in the group of patients who underwent treatment after the lifting of the most severe restrictions (Table 7). The more comorbidities the examined patients had, the more somatic symptoms resulting from being ill they reported.

## 4. Discussion

The current pandemic situation is causing mental-health problems, such as distress, anxiety and depression, both in medical workers and in patients. Due to the nature of dental procedures, during which water-air spray is generated, the risk of coronavirus infection is considered to be very high. The small distance between the doctor and the patient during dental procedures also increases the risk of infection [5,6,19]. Increased levels of anxiety can lead to negligence in attending regular visits and emergency dental appointments, resulting in poor oral health [19,20,21,22,23,24,25]. There was a significant association between patients’ feelings and their willingness to visit the dentist. Patients who regularly visited the dentist before the pandemic are more likely to visit the dental office during the time of COVID-19-related restrictions [11,12].

The patients whose treatments were carried out during the pandemic were characterized by a significantly higher level of ED-5Q index. Our study shows that respondents operated on after the outbreak of the pandemic assessed the quality of their health as significantly worse than those operated on earlier. There is no other published study that compares the quality of surgical patients’ health before and during the pandemic using the EQ-VAS questionnaire; therefore, there is no published data with which to compare our results.

Torales et al. observed an increased percentage of people with anxiety, depression, fear and sleep problems during the COVID-19 pandemic, both in the healthy population and in people with the previously mentioned symptoms [26]. A study from China showed that 53% of people experienced feelings of anxiety and fear about the spreading pandemic [27,28]. The COVID-19 pandemic has exacerbated anxiety levels in many people. Our study shows that low, moderate and high levels of anxiety increased after the outbreak of SARS-CoV-2. The results of our study also show that the pandemic has an impact on the psychological functioning of patients.

Our study shows that the more comorbidities the examined patients have, the more somatic and invalidating symptoms resulting from being ill they reported. Public Health England suggest that patients with comorbidities such as cardiovascular diseases, diabetes, chronic respiratory diseases, hypertension and neoplastic diseases have a higher mortality rate than patients without comorbidities [29].

Although the present study reports on the important issue of oral surgery healthcare during the pandemic, the study limitations must be emphasized. The results represented a single-center experience and were obtained from a small population size. Moreover, dental anxiety is multifactorial, and this research did not explore the effects of personal traits and family-related issues, including socioeconomic status and the level of education of patients [30,31]. The study was not designed to delve into explaining the exact reasons for the observed anxiety levels in examined patients. After the outbreak of the pandemic, only patients with dental emergencies were admitted to the clinic, whereas before the outbreak, planned surgeries were performed. The reason for admittance may also be of importance when assessing anxiety levels in patients, which suggests a possible bias.

## 5. Conclusions

As the COVID-19 pandemic continues to spread, our findings will provide vital guidance for the development of a psychological support strategy for dental surgery patients. It is important to prepare medical staff for the necessity of a special approach towards patients during times of wide-spread coronavirus transmission. The conducted research clearly shows that the number of patients signaling anxiety related to a surgical visit has increased. The study was conducted preliminarily, before the peak of transmission occurred in Poland. Dentists do not usually screen the patients for dental anxiety. The practitioners who are interested in treating patients with dental anxiety should use a screening method to evaluate their patients’ level of anxiety before the procedure. These data will help to perform oral surgery more efficiently, without burdening patients with additional anxiety. Good communication with a trusted dentist, continuity of treatment and regular dental visits, and exposure to a dental environment are the best methods for managing dental fear.

## Figures and Tables

**Table 1:** Characteristics of all 175 patients completing the questionnaire, divided into three groups according to the time of admission. N-number of patients; (%)-per cent of respondents; M-mean, SD-standard deviation; t-value of the Student’s *t*-test; df-degrees of freedom; *p*-level of statistical significance.

		Overall Population Characteristics (N)/(%)	Before Pandemic Population Characteristics (N)/(%)	During Pandemic with High Restrictions Population Characteristics (N)/(%)	During Pandemic When the High Restrictions Were Lifted Population Characteristics (N)/(%)	χ^2^	df	*p*-Value
**Group**		175 (100.0)	47 (26.9)	57 (32.55)	71 (40.55)			
**Age**	18-35	89 (50.9)	30 (33.7)	24 (27.0)	35 (39.3)	7.089563	4	0.13123
	36-55	51 (29.1)	13 (25.5)	18 (35.3)	20 (39.2)			
	56-85	35 (20.0)	4 (11.4)	15 (42.9)	16 (45.7)			
**Sex**	Female	108 (61.7)	29 (26.8)	30 (27.8)	49 (45.4)	3.591385	2	0.16601
	Male	67 (38.3)	18 (26.9)	27 (40.3)	22 (32.8)			
**Place of residence**	<10.000	45 (25.7)	13 (28.9)	16 (35.55)	16 (35.55)	13.59576	6	0.03449
	10.000–50.000	28 (16.0)	4 (14.3)	5 (17.9)	19 (67.8)	Fi = 0.28		
	50.000–100.000	30 (17.1)	12 (40.0)	11 (36.7)	7 (23.3)			
	>100.000	72 (41.1)	18 (25.0)	25 (34.7)	29 (40.3)			
**Type of performed surgery**	Tooth extraction	121 (69.1)	21 (17.36)	47 (38.84)	53 (43.80)	18.92567	2	0.00008
	Surgical tooth extraction	54 (30.9)	26 (48.15)	10 (18.52)	18 (33.33)	Fi = 0.33		
	Others	0	0	0	5 (38.5)			
**Amount of removed teeth**	One	146 (75.4)	46 (31.50)	44 (30.14)	56 (38.36)	9.761003	2	0.00759
	More than one	29 (16.6)	1 (3.45)	13 (44.83)	15 (51.72)	Fi = 0.24		
**Comorbidities**	None	117 (66.9)	31 (26.5)	38 (32.5)	48 (41.0)	4.412333	6	0.62106
	One	41 (23.4)	14 (34.1)	13 (31.7)	14 (34.2)			
	Two	16 (9.1)	2 (12.5)	6 (37.5)	8 (50.0)			
	More than two	1 (0.6)	0 (0.0)	0 (0.0)	1 (100.0)			

**Table 2:** Results of the COVID-19 questionnaire in patients treated after the outbreak of SARS-CoV-2 according to the time of admittance. N-number of patients; (%)-per cent of respondents; M-mean, SD-standard deviation; *t*-value of the Student’s *t*-test; df-degrees of freedom; *p*-level of statistical significance.

COVID-19 Questionnaire	Number of Patients Who Chose the Affirmative Answer (N)/(%) in the Group of 128 Patients, (M/SD)	Number of Patients Who Chose the Affirmative Answer (N)/(%) in the Group of 57 Patients, during Severe Restrictions, (M/SD)	Number of Patients Who Chose the Affirmative Answer (N)/(%) in the Group of 71 Patients, When the Restrictions Were Lifted, (M/SD)	χ^2^	df	*p*-Value
1. I am concerned about the outbreak of the coronavirus pandemic.	45 (35.2) (2.78/1.23)	21 (36.8) (2.86/1.23)	24 (33.8) (2.72/1.23)	0.1281139	1	0.72040
2. I take precautions to prevent infection, e.g., washing hands, avoiding touching door handles, avoiding contact with people	109 (85.2) (4.27/1.08)	48 (84.2) (4.32/0.95)	61 (85.9) (4.24/1.19)	0.0531619	1	0.81765
3. I follow all the Coronavirus news.	64 (50) (3.04/1.36)	26 (45.6) (3.05/1.30)	38 (53.5) (3.03/1.41)	0.2846553	1	0.59367
4. I have acquired supplies to prepare for the potential consequences of a pandemic.	43 (33.6) (2.63/1.37)	18 (31.6) (2.540/1.36)	25 (35.2) (2.70/1.39)	0.1869924	1	0.66543
5. I believe that this virus is much more dangerous than the seasonal flu.	65 (50.8) (3.32/1.32)	25 (43.9) (3.23/1.28)	40 (56.3) (3.39/1.35)	1.969725	1	0.16048
6. I am concerned that friends or family will be infected.	76 (59.4) (3.47/1.32)	39 (68.4) (3.74/1.22)	37 (52.1) (3.25/1.36)	3.486163	1	0.06188
7. I feel safe in a dentist’s office when I see a high level of medical staff protection.	107 (83.6) (4.19/1.11)	49 (86.0) (4.32/0.87)	58 (81.7) (4.08/1.27)	0.4212752	1	0.51630
8. Before going to the dentist’s appointment, I tried to cope with pain using home methods.	88 (68.9) (3.65/1.39)	46 (80.7) (3.98/1.17)	42 (59.2) (3.39/1.49)	4.973704	1	0.02574
9. Due to the current situation, related to the coronavirus, a dental visit makes me feel more anxious than before.	28 (21.9) (2.31/1.19)	14 (24.6) (2.32/1.31)	14 (19.7) (2.31/1.10)	0.4339412	1	0.51006
10. I believe that it is necessary to provide medicals with overalls, masks and helmets during a pandemic.	112 (87.5) (4.39/0.93)	46 (80.1) (4.32/1.05)	66 (93.0) (4.46/0.82)	4.342123	1	0.03718

**Table 3:** Statistical analysis of the COVID-19 questionnaire in all patients treated after the outbreak of the SARS-CoV-2 pandemic. N—number of patients; M–mean; SD—standard deviation.

COVID	N	Mean	Median	Minimum	Maximum	SD	Skewnes	Kurtosis
Q1	128	2.781250	3.000000	1.000000	5.000000	1.229157	0.16910	−1.13026
Q2	128	4.273438	5.000000	1.000000	5.000000	1.084717	−1.76863	2.60379
Q3	128	3.039063	3.500000	1.000000	5.000000	1.359726	−0.16712	−1.35985
Q4	128	2.632813	2.000000	1.000000	5.000000	1.373948	0.37449	−1.21185
Q5	128	3.320313	4.000000	1.000000	5.000000	1.315765	−0.35988	−0.99105
Q6	128	3.468750	4.000000	1.000000	5.000000	1.315788	−0.47889	−1.01309
Q7	128	4.187500	4.500000	0.000000	5.000000	1.113624	−1.76906	2.96985
Q8	128	3.656250	4.000000	1.000000	5.000000	1.388578	−0.84930	−0.58150
Q9	128	2.312500	2.000000	1.000000	5.000000	1.195464	0.58027	−0.77846
Q10	128	4.398437	5.000000	1.000000	5.000000	0.933538	−1.94076	3.98116

**Table 4:** Comparison of patients operated on during severe restrictions with patients operated on after the lifting of restrictions in terms of the total score from the COVID-19 questionnaire. N-number of patients; M-mean; SD-standard deviation; t-value of the Student’s *t*-test; df-degrees of freedom; *p*-level of statistical significance.

COVID-19	During Severe Restrictions			After Lifting the Restrictions			*t*-Test		
	*N*	*M*	*SD*	*N*	*M*	*SD*	*t*	*df*	*p*-Value
Total Score	57	34.67	6.71	71	33.59	7.67	0.83	126	0.407

**Table 5:** Statistical analysis of COVID-19 questionnaire results in patients treated after the SARS-CoV-2 pandemic according to the time of admittance. N-number of patients; R-average rank; *p*-level of statistical significance.

COVID-19	During Severe Restrictions			After the Restrictions Were Lifted			Statistical Analysis	
	*N*	*R*	*Me*	*N*	*R*	*Me*	*Z*	*p*-Value
Q1	57	66.82	68.99	71	62.64	66.07	0.63	0.528
Q2	57	63.96	68.00	71	64.93	65.19	−0.14	0.886
Q3	57	65.07	3.00	71	64.04	3.00	0.15	0.878
Q4	57	62.20	4.00	71	66.35	4.00	−0.63	0.532
Q5	57	61.50	4.00	71	66.91	3.00	−0.82	0.414
Q6	57	71.66	1.00	71	58.75	1.00	1.95	0.051
Q7	57	66.12	4.00	71	63.20	4.00	0.44	0.659
Q8	57	72.36	1.64	71	58.19	1.71	2.15	0.032
Q9	57	63.33	0.00	71	65.44	0.00	−0.32	0.752
Q10	57	63.52	0.00	71	65.29	0.00	−0.27	0.790

**Table 6:** Statistical results of MDAS, ED-5Q and EQ-VAS questionnaire according to the time of admittance. N-number of patients; M–mean; SD-standard deviation; H-the Kruskal-Wallis H test; *p*-level of statistical significance.

Result	Group	*N*	*M*	*Me*	*Min*	*Max*	*SD*	*Skewnes*	*Kurtosis*
MDAS	Before pandemic	47	11.17	10.00	5.00	24.00	5.02	0.52	−0.54
	During severe restrictions	57	11.42	10.00	5.00	25.00	5.35	0.79	0.00
	After lifting the restrictions	71	11.61	11.00	5.00	23.00	4.66	0.35	−0.83
H(2.175) = 0.48; *p* = 0.787									
ED-5Q	Before pandemic	47	6.70	6.00	5.00	13.00	1.92	1.48	2.27
	During severe restrictions	57	7.54	7.00	5.00	19.00	2.41	2.15	8.08
	After lifting the restrictions	71	7.51	7.00	5.00	16.00	2.24	1.29	2.41
H(2.175) = 6.11; *p* = 0.047									
EQ-VAS	Before pandemic	47	82.79	85.00	40.00	100.00	14.49	−1.27	1.381
	During severe restrictions	57	72.68	80.00	20.00	100.00	18.91	−0.871	0.420
	After lifting the restrictions	71	77.63	80.00	30.00	100.00	16.26	−0.706	−0.047
H(2.175) = 8.85; *p* = 0.0119									

**Table 7:** Questionnaire results and comorbidities according to the time of admittance. N-group size; R-value of Spearman’s *R* test; *p*-level of statistical significance.

Comorbidities	All Respondents			Before the Pandemic			During Severe Restrictions			After Lifting the Restrictions		
	N	R	*p*-Value	N	R	*p*-Value	N	R	*p*-Value	N	R	*p*-Value
MDAS	175	0.063	0.4	47	0.116	0.433	57	0.011	0.929	71	0.071	0.556
Q1	128	0.22	0.012	0	0	0	57	0.258	0.051	71	0.187	0.117
Q2	128	−0.061	0.488	0	0	0	57	−0.019	0.884	71	−0.09	0.45
Q3	128	0.121	0.17	0	0	0	57	0.256	0.054	71	0.026	0.826
Q4	128	0.108	0.221	0	0	0	57	0.024	0.855	71	0.177	0.137
Q5	128	0.192	0.029	0	0	0	57	0.285	0.031	71	0.127	0.288
Q6	128	0.106	0.23	0	0	0	57	0.203	0.128	71	0.032	0.79
Q7	128	0.04	0.648	0	0	0	57	0.252	0.058	71	−0.11	0.359
Q8	128	−0.052	0.558	0	0	0	57	−0.242	0.068	71	0.076	0.527
Q9	128	−0.054	0.54	0	0	0	57	−0.004	0.974	71	−0.08	0.504
Q10	128	−0.071	0.409	0	0	0	57	0.093	0.487	71	−0.214	0.072
COVID-19 Total Score	128	0.155	0.08	47	0	0	57	0.203	0.129	71	0.111	0.354
ED-5Q	175	0.234	0.002	47	0.163	0.272	57	0.156	0.245	71	0.327	0.005
EQ-VAS	175	−0.09	0.235	47	0.006	0.96	57	−0.051	0.702	71	−0.181	0.13
